# Normative Data for the NeuroCom^®^ Sensory Organization Test in Subjects Aged 80–89 Years

**DOI:** 10.3389/fnhum.2021.761262

**Published:** 2021-11-16

**Authors:** Laura Perucca, Antonio Robecchi Majnardi, Silvia Frau, Stefano Scarano

**Affiliations:** ^1^Department of Neurorehabilitation Sciences, IRCCS Istituto Auxologico Italiano, Milan, Italy; ^2^Department of Biomedical Sciences for Health, University of Milan, Milan, Italy

**Keywords:** normative data, balance, posturography, EquiTest^®^, Sensory Organization Test, aging

## Abstract

Aging is known to increase the risk of falling. In older people, whose share in the total population is rising sharply, the Sensory Organization Test (SOT, Equitest NeuroCom) is a useful tool during rehabilitation and in clinical research for assessing postural stability, risk of falling, and balance improvement. Normative data for the SOT in the healthy population older than 79 years have not been previously published. We recruited 53 recreationally active healthy subjects aged 80 years and older from the general population in a cross-sectional study. We presented the normative data for SOT for the 80–84 and 85–89 years groups. Our results showed that the “vestibular” balance control tended to be affected by aging more than the vision and proprioception-based systems. A striking reduction in performance after the age of 85 years was observed. These findings will be useful for clinical and research purposes.

## Introduction

Humankind is experiencing constant lengthening in life and healthy years. In recent decades, the percentage of the older population has been rising sharply. According to data from the World Population Prospect (2019 Revision), the number of people aged 80 years or older tripled between 1990 and 2019, growing up to 143 million, and this number will triple by 2050, reaching 426 million (United Nations Department of Economic and Social Affairs [UNDESA], 2020). The World Health Organization defines an older person as one with a chronological age of 65 years or older (World Health Organization [WHO], 2013). Various subgroupings have been proposed by gerontologists to distinguish the diversity of older age (Forman et al., 1992; Zizza et al., 2009). The simplest criteria distinguish young old (60–69 years), middle old (70–79 years), and very old (80+ years) (Forman et al., 1992). This subdivision is further articulated by defining the oldest old (OO) as those aged 85+ years (Garfein and Herzog, 1995), thus implicitly limiting the very old (VO) to a 5-year wide group. However, these definitions are frequently updated as life expectancy continues to rise. The old population is the most susceptible to illness and disability (Christensen et al., 2009): the risk of falling increases with aging and is a major risk after 85 years old (Ferrer et al., 2012), becoming one of the main problems associated with aging (Rogers and Mille, 2003).

In physical and rehabilitation medicine, a large variety of clinical and instrumental tests are used to evaluate postural stability, that is, the ability to control the position of the center of mass in relation to a person’s base of support, during either static or dynamic tasks. The computed dynamic posturographic EquiTest^®^ System (NeuroCom^®^ International, Inc., Clackamas, OR, United States) (NeuroCom^®^ International Inc., 2003) provides qualitative and quantitative analysis of balance control performance through the description of the center of gravity (COG) sway during standing (Nashner and Peters, 1990). Within the EquiTest^®^ System, the Sensory Organization Test (SOT) creates sensory-conflict conditions, making the visual and/or the proprioceptive balance control systems unreliable. Scores are assigned to the oscillation of the subject (the higher, the greater the stability). In essence, the reliable system(s), including the vestibular one, are stressed in order to substitute the unreliable ones. Based on the SOT scores in the various conditions, “sensory analysis” (SA) scores are provided. SOT and SA norms are provided for age groups up to 79 years (20–59, 60–69, 70–79) (NeuroCom^®^ International Inc., 2003). Those for subjects aged 80+ years are lacking.

From a quantitative point of view, normative data are crucial in the understanding of balance performance in healthy subjects (Guskiewicz et al., 1997; Ferber-Viart et al., 2007), in patients affected by vestibular (Pedalini et al., 2009; Alahmari et al., 2014) or other neurological disorders (Voorhees, 1990), and in the aging population as such (Anacker and Di Fabio, 1992; Buatois et al., 2007). Since healthy elderly people are known to show greater body sways during balance tasks with respect to younger individuals (Murray et al., 1975; Forth et al., 2007), a tool to assess whether these augmented oscillations have to be considered physiological or not is necessary, for both clinical and research purposes. The SOT has shown a decline in performance with increasing age in healthy adults (Forth et al., 2007). This study aimed to provide normative data for EquiTest SOT and SA for VO (aged 80–84 years) and OO subjects, with the latter group defined for this study in the 85–89 years range.

## Materials and Methods

### Participants

Subjects aged 80+ years were recruited from a general healthy Italian population. To be included, subjects older than 79 years old and younger than 90 years old had to be able to walk without aid for at least 50 m, provide informed consent, and understand the instructions given during clinical and instrumental evaluations. We excluded individuals with diseases affecting the nervous system, both central and/or peripheral (e.g., Parkinson’s disease, previous traumatic brain injury, pallesthesia < 4/8), previous hip or knee joint replacement or other major surgeries or pathologies affecting the lower limbs or the spine, and acute or chronic pain. Mild visual impairment and mild hearing loss that did not affect activities of daily living were not considered as exclusion criteria. We investigated medication intake to rule out interactions with balance. All participants underwent a standardized neurological examination and structured medical interviews.

The study enrolled 53 subjects fitting the inclusion/exclusion criteria for T0 evaluation. We performed the second evaluation (T1) 2 weeks after T0 to assess the stability and reproducibility of the measurements. Of the 53 subjects, 39 carried out both test sessions. 14 participants dropped out after performing T0 evaluation, for the following reasons: *n* = 2 nausea or dizziness occurred after T0, *n* = 1 gym exercises performed between T0 and T1, *n* = 4 onset of acute pathologies after T0, *n* = 3 reported the testing session as too tiring and refused to participate to the follow-up, and *n* = 4 unsuitable climatic conditions at T1 date (hot summer temperatures).

Motor and cognitive status of the sample was assessed using validated instruments for the main functional domains: Mini Mental State Examination adjusted for age and schooling for cognitive status (Folstein et al., 1975; Magni et al., 1996), Functional Independence Measure for the overall degree of motor and cognitive independence (Linacre et al., 1994), 10-m Walking Test for gait (Brandstater et al., 1983), Equiscale (Tesio et al., 1997), and Dizziness Handicap Inventory short form (Tesio et al., 1999) for balance.

All tests were conducted at the Department of Neurorehabilitation of the Istituto Auxologico Italiano Research Hospital (Milan, Italy). All subjects were informed of the testing procedures and signed a written consent form approved by the hospital’s ethics committee, which also approved the study. This trial has been registered at ClinicalTrials.gov (Identifier: NCT04773496).

To assess postural stability, we used an EquiTest System equipped with the Data Acquisition Toolkit (software version 8.6.1). Data on patients’ age and height (measured on a precision scale) were provided at the beginning of the test as required by the device software. The EquiTest uses a mobile visual surrounding and a support surface containing a dual-force plate, consisting of two 23 × 46 cm footplates connected by a pin joint that can rotate in the sagittal plane following the patient’s anteroposterior sway, thus systematically altering the visual and somatosensory environments. We asked the subjects to stand upright on the instrument support without wearing footwear, with foot placement standardized relative to their height and with arms relaxed at their sides. They had to look straight forward and stand as still as possible during the trials. The SOT measures the sway of the COG under six different conditions (Table 1). The visual surround and the force plate, separately or together, may move in a sway-referenced manner, following the patient’s own anteroposterior sway, thus delivering inaccurate information to the eyes, feet, and joints. Subjects wore a safety jacket hanging from the top of the instrument frame. Data were recorded during three trials for each condition. Each trial lasted 20 s. The sway gain setting was fixed at 1.00, making the support surface and/or visual surround matching the patient’s sway exactly.

**TABLE 1:** The six conditions of the Sensory Organization Test [Adapted from Instructions for Use: EQUITEST^®^ SYSTEM OPERATOR’S MANUAL, Clackamas, OR (United States): NeuroCom^®^ International Inc., 2003].

	Condition 1	Condition 2	Condition 3	Condition 4	Condition 5	Condition 6
Eyes	Open	Closed	Open	Open	Closed	Open
Surroundings	Fixed	n.a.	Sway-referenced	Fixed	n.a.	Sway-referenced
Platform	Fixed	Fixed	Fixed	Sway-referenced	Sway-referenced	Sway-referenced
Sensory System used	Somatosensory	Somatosensory	Somatosensory	Vision	Vestibular	Vestibular

*n.a., not applicable.*

In the EquiTest System the amount of sway of the COG is expressed in degrees to allow comparison of scores between individuals of different heights. The angular limits of stability are nearly the same for all normal adults, regardless of height (NeuroCom^®^ International Inc., 2003). The equilibrium score compares the patient’s anteroposterior (AP) sway during each trial to a theoretical sway stability limit of 12.5 degrees (NeuroCom^®^ International Inc., 2003). SOT Scores for each condition are given in the range of 0–100, with higher scores representing better performance and participants swaying to the limits of stability receiving low scores (NeuroCom^®^ International Inc., 2003). Score 0 is assigned to a trial marked as “fall” (a stepping reaction, hands touching the surround or, in extreme cases, subject being supported by the safety jacket). Score 100 is assigned to full stability (never achievable in practice). A cumulative 0–100 SOT score (composite SOT) is also assigned to the overall test. Normative data are given as age-specific 95th percentile limits.

The SA scores (0–100, the higher the better the condition) were computed as ratio between the mean scores of specific conditions: SOM represents the ratio of the second to the first condition (if reduced it suggests a somatosensory impairment is present); VIS is the ratio of the fourth condition to the first (if reduced i.e., inability to use vision for compensatory purposes); VEST is the ratio of the fifth to the first condition (if reduced i.e., a possible vestibular deficit as in condition 5 balance is entrusted exclusively to the vestibular system). PREF highlights the visual preference (ratio of conditions with unreliable vision compared to those where vision is absent—i.e., conditions (3 + 6) to (2 + 5), when reduced it implies the subject relies on visual information even when those are unreliable (NeuroCom^®^ International Inc., 2003).

The first evaluation (T0) (subject’s history, functional scales, and instrumental evaluation) took approximately 90 min for each subject. To assess the reliability of the measurement, we repeated all six conditions of the SOT (T1) 2 weeks later (± 2 days). We asked the participants not to train or practice any kind of exercise between T0 and T1. Data collection was carried out by operators with experience in this assessment or trained for this purpose.

### Statistical Analysis

We performed data analysis using STATA statistical software, version 11 (Computing Resource Center, Los Angeles, CA, United States, 2011). Descriptive statistics (mean ± SD, median) for each SOT condition and composite scores were generated. The SOT’s score distributions for the entire sample and for the two different populations (VO and OO) were tested for normality (skewness and kurtosis test). Data are represented using bar charts and box plots. To allow comparison with the normative data of the previous 10-year age group, we set the level of significance at *p* < 0.05. We calculated the normative data as stated by NeuroCom, setting the limits of normality at the 5th percentile by applying the following equation: *SOT norms* = *mean - (SD ^∗^ 1.67)* (NeuroCom^®^ International Inc., 2003).

To assess the test-retest reliability, we used intraclass correlation coefficients (ICC 3.1, two-way random-effect model, absolute agreement) for the composite SOT and for each condition evaluated at T0 and T1. According to the literature (Koo and Li, 2016), reproducibility is labeled as poor when ICC < 0.5, moderate if between 0.5 and 0.75, good if between 0.75 and 0.9, excellent when > 0.90.

## Results

53 subjects participated in the study (25 women, 47%). The mean ± SD age of the entire sample was 83.4 ± 3 years (median: 82 years), with a range of 80–89 years. The mean and median ages did not differ substantially between men (83 ± 3.2 years, median: 81.5 years, range: 80–89 years) and women (83.88 ± 6 years, 84 years, 80–89 years). By applying the stated definition of VO (80–84 years) and OO (for this study 85–89 years), we divided the sample into two populations, as follows: VO, *n* = 35 (66%), and OO, *n* = 18 (34%). For both VO and OO, descriptive statistics for demographics and functional tests are provided in Table 2. The subjects were cognitively intact and had no balance, walking, or global functional impairments. T1 assessment was performed by 26 VO and 13 OO participants.

**TABLE 2:** Descriptive data on very old (VO) and oldest old (OO) groups.

		VO (80–84 years old) (*n* = 35)	OO (85–89 years old) (*n* = 18)
	Score range (better score)	Mean ± SD (Median)	Mean ± SD (Median)
Age	n.a.	81.5 ± 1.4 (81)	87.1 ± 1.7 (87)
Male	n.a.	57.1% (*n* = 20)	44.4% (*n* = 8)
Number of medications	n.a.	2.5 ± 2.3 (2)	3.0 ± 2 (3)
MMSE(*)	0–30 (30)	28.6 ± 1.8 (29.1)	29.6 ± 2.4 (30.3)
FIM^®^—Comprehensive	18–126 (126)	125 ± 1.4 (126)	123.9 ± 2.4 (124)
FIM^®^—Motor	13–91 (91)	90.1 ± 1.4 (91)	89.6 ± 0.8 (90)
FIM^®^—Cognitive	5–35 (35)	35 ± 0.16 (35)	34.3 ± 2.4 (35)
Equiscale	0–16 (16)	15.3 ± 1 (16)	14.5 ± 1 (15)
DHI	0–13 (0)	0.6 ± 0.94 (0)	0.8 ± 0.8 (1)
Step length cm	Ability to perform the 10 mWT	64.9 ± 7.6 (65)	57.5 ± 6.9 (58)
Gait speed m/s		1.17 ± 0.2 (1.18)	1.1 ± 0.1 (1.1)

*n.a., not applicable; MMSE, Mini Mental State Examination; FIM, Functional Independence Measure; DHI, Dizziness Handicap Inventory short form; 10 mWT, 10-m Walking Test.*

(*) MMSE scores have been corrected for age and schooling. The number of medications taken by subjects ranged from 0 to 10 and were mostly in the three main categories of anti-hypertensives, proton-pump inhibitors, and those for benign prostate hypertrophy. Among the subjects, 17% (n = 9) did not take any medication, 17% (n = 9) took only one medication, and 34% (n = 18) took a maximum of two or three drugs per day. Only four subjects used more than six medications per day.

The SOT scores for the entire sample and for the two different groups (VO and OO) were not normally distributed, according to the skewness and kurtosis test. The bimodal distribution of the observed scores seemed to fit the study’s originally proposed division between two different populations (VO and OO). The non-parametric Mann–Whitney test confirmed that the scores for each condition (except conditions 3 and 6) and composite SOT were statistically different between the two groups.

**FIGURE 1:**
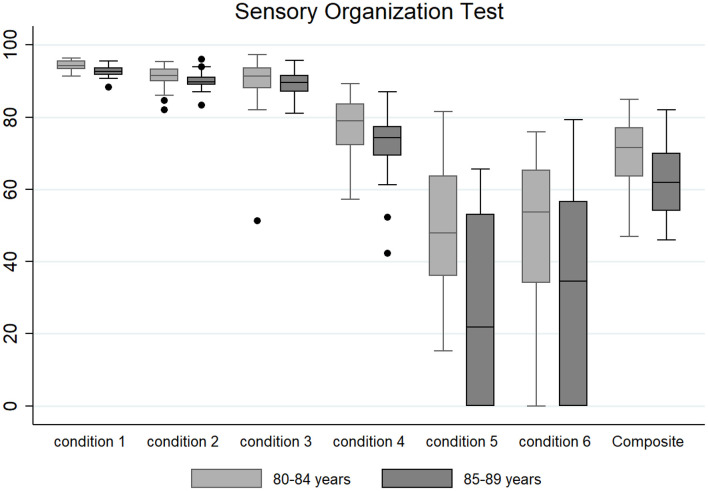
Box plots (median and interquartile range; outliers are represented as dots) for the six testing conditions and for the composite Sensory Organization Test for the very old (80–84 years) and oldest old (85–89 years) groups.

**FIGURE 2:**
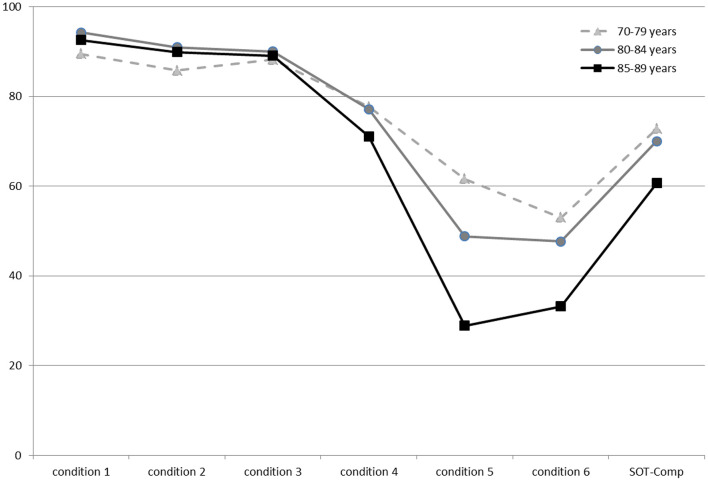
Mean values for the three age groups in the six conditions and for the composite Sensory Organization Test data. The lowest values in condition 5 describe an age-related worsening of vestibular function. Data for the 70–79 age group are provided by NeuroCom^®^ International Inc. (2003).

**FIGURE 3:**
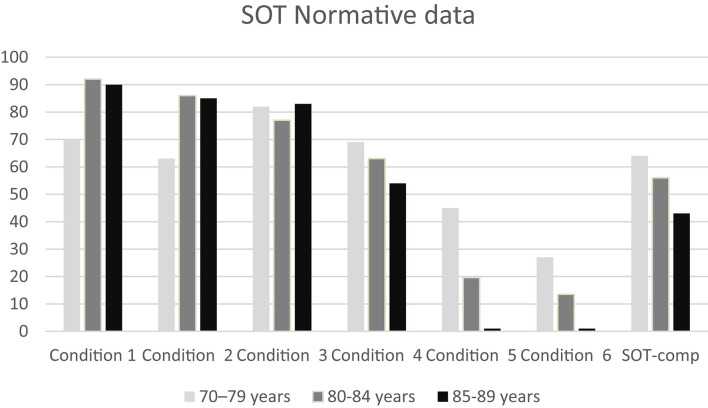
Normative data for the six conditions and for the composite Sensory Organization Test data in the three age groups considered in this study. The striking reduction in norm values in conditions 5 and 6 is due to the wide standard deviation in those conditions. Data for the 70–79 age group are provided by NeuroCom^®^ International Inc. (2003).

**FIGURE 4:**
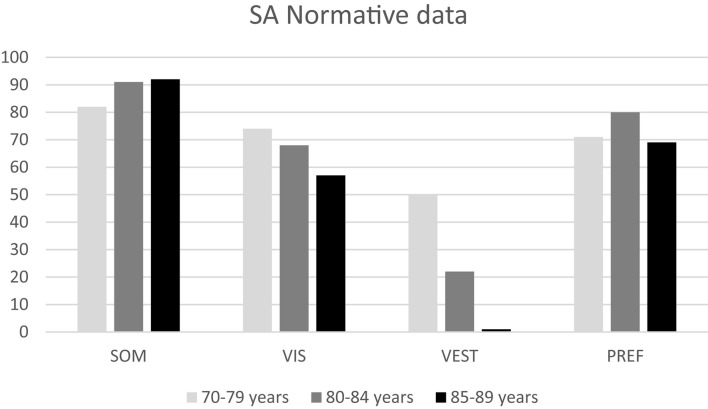
Normative data for sensory analysis (SA) for the three age groups considered in this study. Data for the 70–79 age group are provided by NeuroCom^®^ International Inc. (2003).

Descriptive data (median and interquartile range) for the six conditions and the composite SOT for the two groups are shown as box plots in Figure 1, while mean scores for each condition and for the composite SOT are presented in Figure 2. Table 3 summarizes the descriptive statistics (mean ± SD and median), normative data, and variance analysis for SOT. Descriptive statistics (mean ± SD and median) and normative data for SA are presented in Table 4. Normative data for each condition and composite SOT are reported in Figure 3, while normative data for SA are in Figure 4. In Tables 3, 4 and Figures 2–4 the data on the 70–79 years age group, as provided by NeuroCom in the User’s manual, are reported in order to give the reader a quick comparison among the different age groups. The NeuroCom’s sample was composed of 29 participants (14 women and 15 men). No further statistical analysis has been performed on the NeuroCom’s sample data.

**TABLE 3:** Descriptive, variance analysis, and normative data for each condition and composite SOT for three age groups: 70–79 years (data provided by NeuroCom^®^ International Inc., 2003), very old (VO, 80–84 years), and oldest old (OO, 85–89 years).

Age group	70–79 years	VO 80–84 years	OO 85–89 years	VO vs. OO	70–79 years	VO	OO
					
	Mean ± SD	Mean ± SD (Median)	Mean ± SD (Median)	Variance analysis	SOT normative data		
Condition 1	89.4 ± 11.4	94.3 ± 1.3 (94.3)	92.6 ± 1.7 (92.6)	*p* < 0.000	70	92	90
Condition 2	85.8 ± 13.5	91.1 ± 3 (91.6)	89.9 ± 2.7 (89.8)	*p* < 0.048	63	86	85
Condition 3	88.2 ± 3.8	90 ± 7.6 (91.3)	89.1 ± 3.4 (89.6)	*p* < 0.098	82	77	83
Condition 4	77.6 ± 5.5	77.2 ± 8.3 (79.0)	71.2 ± 10.4 (74.3)	*p* < 0.018	69	63	54
Condition 5	61.6 ± 10.2	48.8 ± 17.5 (48.0)	28.9 ± 24.6 (21.8)	*p* < 0.005	45	20	0
Condition 6	53.0 ± 15.5	47.7 ± 20.5 (53.6)	33.2 ± 27.3 (34.6)	*p* < 0.064	27	13	0
Composite SOT	72.8 ± 5.4	69.9 ± 8.4 (73.0)	60.7 ± 10.3 (58.5)	*p* < 0.003	64	56	43

*SOT, Sensory Organization Test.*

**TABLE 4:** Sensory analysis (SA) descriptive (mean ± SD, median) and normative data for three age groups: 70–79 years (data provided by NeuroCom^®^ International Inc., 2003), very old (VO, 80–84 years), and oldest old (OO, 85–89 years).

	70−−79years	VO		OO		SA normative data		

	Mean ± SD	Mean ± SD	Median	Mean ± SD	Median	70–79 years	VO	OO
SOM	95.1 ± 7.9	96.5 ± 3	97	97.1 ± 3.1	97	82	91	92
VIS	85 ± 0.066	81.8 ± 8	82	76.9 ± 11.7	80	74	68	57
VEST	67.3 ± 10.4	51.8 ± 18	52	31.4 ± 27	23	50	22	0
PREF	94.9 ± 14.4	98.5 ± 11	100	105.1 ± 21.8	101	71	80	69

*SOM, somatosensory; VIS, visual; VEST, vestibular; PREF, visual preference.*

We tested the test-retest reliability of our sample and the ICC values provided good reproducibility of the Composite SOT and moderate to good reproducibility for each of the six SOT condition scores. ICC values are reported in Table 5.

**TABLE 5:** Interclass correlation coefficients (ICC) for each condition and for composite SOT in the whole sample between T0 and T1.

	ICC
Condition 1	0.52
Condition 2	0.79
Condition 3	0.55
Condition 4	0.73
Condition 5	0.71
Condition 6	0.67
composite SOT	0.76

*SOT, Sensory Organization Test.*

## Discussion

The study of SOT in the healthy population has recently developed (Guskiewicz et al., 1997; Ferber-Viart et al., 2007; Pierchała et al., 2012; Pletcher et al., 2017), but not yet in the population aged 80–89 years. As suggested by the increased incidence of falls in the elderly, one or more of the systems involved in postural control lose efficiency with age (Peterka and Black, 1990). Our results showed that balance control in VO and OO is less effective when vestibular information is the only one available, in particular above 84 years of age. This finding is consistent with previous clinical observations (Ferrer et al., 2012). The older adults of the third millennium are the healthiest, most active, and longest-living in human history and are now a demographic phenomenon in most countries (United Nations Department of Economic and Social Affairs [UNDESA], 2020). The literature deals comprehensively with the concept of healthy aging and the interaction between physiological age-related deficits and actual neurological pathology (Morris and McManus, 1991; Kaye et al., 1994; Nardone et al., 2000; Papegaaij et al., 2014). Aging involves the degeneration of various central nervous system (CNS) and peripheral nervous system (PNS) structures, with a loss in brain volume and weight around 5% per decade after age 40 and possible more above age 70 (Peters, 2006), and physiological changes in postural control and balance (Nardone et al., 2000; Kaye et al., 1994; Camicioli et al., 1997), related to sensory deficits (Judge et al., 1995), CNS integration abnormalities (Morris and McManus, 1991), and musculoskeletal aging (Judge et al., 1996). From this point of view, a reduction in neurological performance can be defined as a sequela of neuroanatomical and neurofunctional changes (Morris and McManus, 1991; Papegaaij et al., 2014). The risk of falling increases in older adults, and balance impairment is one of the main targets of rehabilitation treatment (Chang et al., 2004).

As expected, mean values decreased in both VO and OO groups from Condition 1 (i.e., all sensory information available) to Condition 5 (i.e., visual information absent and proprioception not reliable). In the variance analysis, we observed that the scores for all conditions and for the composite SOT were statistically different between the VO and OO, with the exception of condition 6 and, broadly, of condition 3. A similar observation for condition 3 has already been provided in a previous study (Pletcher et al., 2017). In that paper statistical differences were seen between military groups with different tactical demands in all SOT conditions with the exception of condition 3; the authors hypothesized that the similar distribution of the SOT scores for condition 3 and somatosensory scores among groups might reflect the disadvantage to the visual system due to the sway-referenced surround. In our sample this result may suggest that aging can affect the use of proprioceptive information less than vestibular information with respect to balance control. However, this aspect requires further investigation.

The normative data for conditions 1 and 2 are similar between VO and OO. In the OO group the norms for conditions 5 and 6 are equal to zero, and this evidence may imply that vestibular information are not sufficient for OO for maintaining balance control, and that a low vestibular score at the SOT could be considered as “normal” (whereas it would still be considered as pathological in the VO population).

Moreover, the comparison of normative data for the six SOT conditions, composite SOT and SA for VO and OO with those of the previous decade (Tables 3, 4) suggests some thoughts:

– The normative data for conditions 1 and 2 for the two groups in our sample were higher than those stated by NeuroCom for the 70–79 years age group. The reason for this discrepancy could have various origins. First, the sample adopted by NeuroCom for the 70–79 age group was smaller (*n* = 29, 14 female) than ours. Although declared asymptomatic, their sample was not previously evaluated for functional performance and medication intake. Second, aging for our subjects occurred 20 years later than the NeuroCom original study, so that health care conditions might have improved. Third, subjects who survived for a long time without health concerns could have had a functional advantage during aging. Fourth, the mediterranean environmental context might have fostered a healthier aging.
– The greatest differences with the NeuroCom 70–79 years group can be found for Conditions 5 and 6 norms: both VO and OO display lower values than the 70–79 group. This evidence may suggest that a reduction in the efficiency of the vestibular system can be involved in the increasing rate of falling in the elderly.

The reliability of the measures of SOT scores and of the composite SOT were found to be moderate to good. The reliability of SOT scores for Conditions 1–6 were comparable to those reported in previously published papers for individuals aged 20–80 + (19) and above 65 years of age (Ford-Smith et al., 1995). As already pointed out by Forth et al. (2007), there is a considerable variability in the ICCs of the different SOT conditions, ranging from 0.52 to 0.79 in our sample.

The normative data provided by this work may lead clinicians and researchers in defining the degree of balance impairment in individuals aged 80–89 years old. Moreover, the identification of the impaired sensory systems can guide the development of rehabilitation programs tailored on the patient and the measurement of rehabilitation outcomes. This information, together with other validated tool, will help in assessing the risk of falls in elderly people.

## Conclusion

This study provided normative data for SOT and SA assessed by the EquiTest^®^ System in healthy older subjects. Poor postural stability is a risk factor for falls and subsequent injury. To our knowledge, this study is the first to present SOT and SA norms for subjects aged 80+ years. Moreover, we investigated subjects in the oldest old category (85–89 years). Data from our research can assist both clinicians and researchers who deal with balance in older subjects.

## Limits of the Study

The recorded data for the sample were not normally distributed. The variance analysis revealed how the vestibular performance decreased suddenly over a 5-year horizon (80–84 years vs. 85–89 years) and suggested an opportunity for studies with an older and wider span of older healthy subjects. However, it is not easy to find a large number of healthy subjects aged 85 + years. More studies focused on subjects aged 85 + or 90 + years will be possible and easier in the future, considering the global phenomenon of aging.

## Data Availability Statement

The datasets presented in this study can be found in online repositories. The names of the repository/repositories and accession number(s) can be found below: doi: 10.5281/zenodo.5215848.

## Ethics Statement

The studies involving human participants were reviewed and approved by Comitato Etico—Istituto Auxologico Italiano IRCCS. The patients/participants provided their written informed consent to participate in this study.

## Author Contributions

LP designed the study. LP and SF collected the data. LP and AR analyzed the data. AR wrote the manuscript. LP and SS reviewed the manuscript. All authors contributed to the article and approved the submitted version.

## Conflict of Interest

The authors declare that the research was conducted in the absence of any commercial or financial relationships that could be construed as a potential conflict of interest.

## Publisher’s Note

All claims expressed in this article are solely those of the authors and do not necessarily represent those of their affiliated organizations, or those of the publisher, the editors and the reviewers. Any product that may be evaluated in this article, or claim that may be made by its manufacturer, is not guaranteed or endorsed by the publisher.
